# Examining the relationship between preferred music types and temperament-character in individuals diagnosed with alcohol and substance use disorders

**DOI:** 10.1186/s40359-025-03648-2

**Published:** 2025-11-24

**Authors:** Muhammed Raşit Bardakçı, Hasan Kadir Yılmaz, Ahmet Bulent Yazici, M. Nevra Kupana, Esra Yazici

**Affiliations:** 1 Clinic of Psychiatry, Sakarya Training and Research Hospital, Sakarya, Turkey; 2 Faculty of Medicine, Sakarya University, Sakarya, Turkey; 3 Faculty of Medicine, Department of Psychiatry, Sakarya University, Sakarya, Turkey; 4 Department of Musicology, Sakarya University State Conservatory, Sakarya, Turkey

**Keywords:** Addiction, Alcohol Use Disorder, Temperament, Character, Music, Substance Use Disorder, Türkiye

## Abstract

**Background:**

Individuals with Alcohol Use Disorder (AUD) and Substance Use Disorder (SUD) face significant challenges at personal, familial, and social levels. Temperament and character traits play a key role in addictive behaviors, yet their relationship with music preferences in these patients has rarely been examined. This study explores the relationship between temperament-character traits and music preferences in AUD or SUD patients.

**Methods:**

This cross-sectional study included 351 patients diagnosed with AUD or SUD, who were receiving treatment at the Inpatient Detoxification Centre (IDC). All participants completed the Sociodemographic Data Form, Music Preferences Questionnaire (MPQ), Temperament and Character Inventory (TCI), and Addiction Profile Index (API).

**Results:**

The participants were classified into three distinct clusters as determined by cluster analysis. Cluster 1 comprised individuals demonstrating a preference for high-energy and modern styles (*n* = 53), while most subjects (*n* = 246) were included in the cluster characterized by low interest and fantasy arabesque preferences (Cluster 2). Cluster 3 consisted of subjects with broad and balanced preferences (*n* = 52). There were no significant sociodemographic differences among the clusters, but significant variations in personality traits were found, particularly in novelty seeking, reward dependence, and cooperativeness. Alcohol users preferred Classical Turkish music (*p <.005*), while substance users favoured Hip-hop/Rap (*p <.005*) and Techno/Electronic music (*p <.005*). A weak but significant positive correlation was found between Hip-hop/Rap preference and educational status (ρ = 0.179, *p* = .001). Similarly, Classical Turkish Music preference showed a weak positive correlation with age (ρ = 0.263, *p* < .001).

**Conclusions:**

In conclusion, AUD or SUD patients have low interest in music types and limited musical tastes. It has been observed that there is a relationship between these patients’ novelty seeking and cooperativeness traits and their music preferences, and that individuals prefer certain music types more than others, depending on their diagnosis with weak additional associations with demographic factors, particularly age and educational level.

**Trial registration:**

Not applicable.

## Background

AUD and SUD are widespread public health problems all over the world, in which psychosocial, biochemical and genetic factors play a role in its development and continuation. The physical, psychological, social, economic and legal problems caused by AUD or SUD have a negative impact not only on individuals, but also on their families and indirectly on society as a whole [1]. According to the World Drug Report for 2024, published by the United Nations Office on Drugs and Crime (UNODC), approximately 64 million people are reported to have SUD, marking a substantial increase from the 39.5 million SUD cases reported the previous year [2]. Furthermore, the World Health Organization’s (WHO) Global Status Report on Alcohol, also published in 2024, shows that 3.6% of adults consume more than 60 g of alcohol per day on average (heavy drinkers) [3].

Alcohol and substance use pose significant public health challenges and have been consistently linked to various personality traits [4, 5]. A recent systematic review and meta-analysis by Lui et al. (2022) found that, in the context of alcohol consumption, lower levels of conscientiousness and agreeableness are associated with risky or hazardous drinking [6]. Additionally, high levels of neuroticism are connected to negative consequences related to drinking [5]. Complementing these findings, studies utilizing the Big Five personality model across alcohol, nicotine, marijuana, and gambling disorders have consistently identified high neuroticism, low agreeableness, and low conscientiousness as common traits, suggesting a general predisposition toward addictive behaviours [4]. Furthermore, research using Cloninger’s TCI has shown that substance-dependent individuals tend to score higher in novelty seeking and lower in reward dependence, self-directedness, and cooperativeness, offering insights beyond the Big Five framework [7, 8]. Additionally, younger age and higher levels of novelty seeking have been identified as predictors of drug dependence [6]. More specifically, high neuroticism has been associated with prescription drug misuse, high extraversion with cocaine/crack and stimulant use, high openness to experience with marijuana use, and low agreeableness with both cocaine/crack and illicit opioid use [4].

The impact of music on mood has been the subject of extensive and diverse research. Numerous studies have consistently demonstrated that music can alleviate depressive symptoms and influence stress levels across various age groups [9, 10]. Beyond its emotional effects, music has also been shown to interact with personality traits. For example, a study involving 67 participants found that both mood and personality influence the perception of emotional content in music—such as anger, fear, happiness, sadness, and tenderness—indicating a complex and dynamic relationship between these variables [11]. The relationship between music preferences and personality has long attracted scholarly interest. Despite methodological limitations, this area continues to be actively explored [12]. For instance, a study of 145 university students in Poland found that personality traits such as liveliness, social boldness, alertness, openness to change, and extraversion influenced preferences for specific musical elements influenced preferences for specific musical elements such as faster tempo, rhythmic complexity, and major-key tonality [13]. In a large-scale study involving 36,518 participants, researchers found that the Big Five personality traits were associated not only with preferences for 104 different music genres but also with the underlying reasons individuals listened to music [14]. For example, openness to experience predicted a preference for reflective and complex styles such as jazz and classical, whereas extraversion was related to upbeat and energetic genres like pop, dance, and hip-hop [12, 15]. Further expanding on these findings, a cross-cultural study involving more than 3,500 individuals used multiple methods and samples across diverse geographic regions to explore how personality relates to preferences for four major music dimensions: reflective and complex, intense and rebellious, upbeat and conventional, and energetic and rhythmic. Results revealed associations between music preferences and various psychological factors, including personality traits (e.g., openness to experience), political orientation, and cognitive abilities (e.g., verbal IQ) [15]. However, a 2017 meta-analysis of 28 studies concluded that the relationship between personality and music preferences is generally weak, with openness to experience emerging as the most consistently related trait [16]. Complementary findings from a U.S.-based study indicated that individuals with a high need for novelty and stimulation were more likely to prefer genres such as rap, punk, heavy metal, and reggae [17]. Similarly, research conducted in Italy found that among five affective temperament profiles, as conceptualized by Akiskal’s model——hyperthymic, cyclothymic, depressive, anxious, and nervous—individuals with cyclothymic and hyperthymic temperaments showed a stronger preference for rap music, while those with depressive traits showed less interest in it [18]. The relationship between AUD and SUD in the context of music preferences has been a subject of academic inquiry. A recent meta-analysis encompassing 31 studies and a total of 330,652 participants reported small-to-moderate positive correlations between music engagement and substance use behaviours. This comprehensive analysis reveals that different music formats—specifically audio lyrics, music videos, genre preferences, performance venues, nightclubs, and general music media—exhibit positive associations with substance use. However, the strength of these associations differs across the various formats analysed. Moreover, the findings indicate that music genres such as electronica, rap, hip-hop, urban, rock, top charts, pop, reggae, highbrow, and country are also demonstrated positive but varying associations with substance use behaviours. Importantly, preferences for specific music genres differ according to the type of substance consumed, including tobacco, alcohol, and illicit drugs [19]. These insights underscore the complex and correlational interplay between musical engagement and substance use, warranting further investigation in this area of study.

While personality traits in individuals with AUD and SUD have been frequently studied, and the relationship between music preferences and personality traits has been widely examined in general populations, to the best of our knowledge, no research has explored the connection between music preferences and temperament and character traits specifically in individuals with AUD and SUD. Given that temperament and character traits such as novelty seeking, reward dependence, and self-directedness are central to addictive behaviors, considering their potential influence on musical preferences may provide unique insights. This gap in the literature underscores the potential value of examining this relationship within clinical populations.

The aim of the present study is to investigate the relationship between temperament and character traits and music preferences among individuals with AUD and SUD. The study also considers the type of substance used, the severity of dependence, and sociodemographic variables, in order to provide a more nuanced understanding of the factors influencing musical preferences in this population. This study hypothesizes that music preferences are associated with personality traits among individuals with AUD and SUD, and that certain music genres may reflect differences in personality characteristics and addiction-related factors.

## Methods

### Sampling

This prospective study included patients who received either inpatient or outpatient treatment at Sakarya University Training and Research Hospital Inpatient Detoxification Centre (IDC) between November 2024 and April 2025. The study included patients aged 18–65 who voluntarily participated and had been diagnosed with AUD or SUD according to DSM-5 criteria. Patients with known active endocrinological, metabolic, or neurological disorders, as well as those diagnosed with schizophrenia, bipolar disorder, or intellectual disability, were excluded. A power analysis indicated that a minimum of 313 patients was necessary, considering a significance level of 5%, a confidence interval of 80%, and an effect size of 0.20. This effect size is based on previous studies that reported a low-level relationship between music preferences and personality [20]. To ensure adequate representation, we planned to include more participants than the minimum requirement, ultimately enrolling 351 individuals who provided informed consent. Participants were categorized into two groups: alcohol users and substance users, based on their primary substance of choice. Additionally, a clustering analysis was performed to classify patients into three clusters according to their music preferences. After the inpatients completed their withdrawal (one week later) and the outpatients reached clinical stability, they were administered several assessments, including the Sociodemographic Data Form, the MPQ, the TCI, and the API. Ethical approval for the study was obtained from the Sakarya University Faculty of Medicine Health Sciences Scientific Research Ethics Committee on November 19, 2024, with the decision recorded under number E-43012747-050.04-421144-116.

### Materials

#### Sociodemographic data form

The research group developed it to examine patients’ sociodemographic characteristics, including age, gender, educational level, employment status, economic status, marital status, and place of residence.

#### Music preferences questionnaire (MPQ)

This questionnaire, prepared by the research group, includes music genres that are widely listened to in Turkey, music genres from different rhythm groups and well-known music samples from world music. The KONDA (Public Opinion Research and Consulting Limited Company) Music Preferences and Communication Tools report published in 2018 was utilized in the preparation of the survey [21].

#### Addiction profile index (API)

This instrument is a self-report scale comprising 37 items structured across five distinct subscales, developed by Ögel et al. in 2012, designed to evaluate various dimensions and severity of AUD or SUD. This scale, where each item is scored between 0 and 4, yields subscale and total scores by taking the average of the items in each subscale. The subscales assess key aspects including characteristics of substance use, adherence to diagnostic criteria for use disorders, the repercussions of substance use on individual functioning, levels of craving for substances, and the individual’s motivation to discontinue substance use. The comprehensive nature of this scale facilitates a multidimensional understanding of addictive behaviours and their impact on personal and social well-being. This scale, which yielded a Cronbach’s alpha coefficient of 0.89, has been evaluated as a valid and reliable scale [22].

#### Temperament and Character Inventory (TCI)

Cloninger developed a dimensional, psychobiological model of personality that identifies both normal and abnormal traits within two key components: temperament and character. This model consists of 240 items and seven higher-order scales, which are answered as ‘True’ or ‘False’. For all subscales, items scored positively (True = 1, False = 0) and items scored negatively (True = 0, False = 1) are present, and these are summed to obtain scale scores. There are also some items included for control purposes that are not scored at all [23]. In a study conducted by Köse et al. in 2004, the Turkish version of the scale was found to be valid and reliable, with Cronbach’s coefficients ranging from *0.60* to *0.85*. In this framework, the components of temperament highlight individual differences in perception-based habits and skills, which are largely genetically inherited. Temperament is categorized into four main domains: Novelty Seeking (NS), Harm Avoidance (HA), Reward Dependence (RD), and Persistence (P). On the other hand, the components of character develop as a person matures and engage with concepts of self and personal or social activities associated with adulthood. These traits are more culturally influenced and comprise three main domains: Self-Directedness (SD), Cooperativeness (C), and Self-Transcendence (ST) [24].

### Statistical analysis

The data collected for this study were analysed using IBM SPSS Statistics version 25.0. To assess the normal distribution of continuous variables, skewness and kurtosis statistics were evaluated, with values ranging from − 2 to + 2 indicating that the assumptions of normality were satisfied [25]. Categorical variables were analysed using the chi-square test. In cases where significant differences were observed in chi-square analyses involving multiple group comparisons, post hoc tests were conducted. The Bonferroni correction was applied to adjust the reference p-value, and p-values were calculated based on Z-scores. For continuous variables with normal distribution, independent samples t-tests and one-way ANOVA were used to compare group means. When the assumption of normality was violated, the Mann-Whitney U test and the Wilcoxon signed-rank test were employed. Following ANOVA, Tukey’s HSD test was used to adjust p-values for pairwise comparisons, while the Bonferroni correction was applied to control the family-wise error rate in multiple comparisons. Correlation analyses were performed using either Pearson’s r or Spearman’s rank correlation coefficient, depending on the distribution and measurement level of the variables. A significance level of *0.05* was adopted, corresponding to a *95%* confidence interval.

## Results

Patients were categorized into two distinct groups: those diagnosed with AUD and those with SUD. A comparative analysis of sociodemographic characteristics revealed no statistically significant differences between the groups concerning gender, marital status, and economic status (*p* >.05). However, a statistically significant difference was observed in the average age of participants, with the AUD group having a mean age of 41.32 ± 10.94 years, compared to 33.32 ± 8.46 years in the SUD group (t: 6.495; *p* <.001). Regarding educational attainment, the AUD group showed that 47.4% (*n* = 46) had completed primary education, 34.0% (*n* = 33) had obtained a high school diploma, and 18.6% were university graduates. Conversely, in the SUD group, 49.2% (*n* = 125) had completed primary education, 43.3% (*n* = 110) had achieved a high school education, and only 7.5% (*n* = 19) were university graduates. The disparity in educational levels between the two groups was found to be statistically significant (**χ² = 9.702**; *p* =.008) (Table 1).

**Table 1:** Comparison of sociodemographic characteristics according to patients’ diagnoses

		Group		Value	*p*
		Alcohol (*n* = 97)	Substance (*n* = 254)		
**Age**,** Mean ± SD**		41.32 ± 10.94	33.32 ± 8.46	*t = 6.495*	***< 0.001***
**Gender ** **n (%)**	**Female**	33 (%34,0)	79 (%31.1)	*χ²=0.275*	*0.600*
	**Male**	64 (%66.0)	175 (%68.9)		
**Education Status n (%)**	**Primary**	46 (%47.4)	125 (%49.2)	*χ²=9.702*	***0.008***
	**High School**	33 (%34.0)	110 (%43.3)		
	**University**	18 (%18.6)	19 (%7.5)		
**Economic Status** **n (%)**	**< 17k**	41 (%42.3)	112 (%44.1)	*χ²=4.927*	*0.177*
	**17k-34k**	34 (%35.1)	105 (%41.3)		
	**34k-51k**	13 (%13.4)	27 (%10.6)		
	**> 51k**	9 (%9.3)	10 (%3.9)		
**Marital Status** **n (%)**	**Single**	27 (%27.8)	98 (%38.6)	*χ²=3.541*	*0.170*
	**Married**	54 (%55.7)	121 (%47.6)		
	**Divorced**	16 (%16.5)	35 (%13.8)		

Subsequent post hoc analyses utilizing Bonferroni correction indicated a significant difference in the proportions of university graduates between the two groups (*p =.004*), adhering to a significance threshold of *0.008* established through the Bonferroni adjustment.

No statistically significant differences were observed between the AUD group and the SUD group concerning temperament and character dimensions, as assessed by the TCI and the API total scores (*p >.05*). In contrast, significant differences emerged when comparing the groups based on their preferred music genres. The mean score for Classical Turkish Music was 1.97 ± 1.92 in the AUD group, while the SUD group reported a mean score of 1.28 ± 1.58, indicating a statistically significant preference for Classical Turkish Music within the AUD group (t = 3.159; *p* =.002). For Hip-hop/Rap Music, the AUD group’s mean score was 1.42 ± 1.91 compared to the SUD group’s mean score of 2.16 ± 2.07, demonstrating a statistically significant preference for Hip-hop/Rap Music in the SUD group (*t = −3.027; p =.003*). Similarly, regarding Techno Electronic Music, the AUD group reported a mean score of 0.84 ± 1.51, while the SUD group scored 1.42 ± 1.94. This finding suggests a statistically significant preference for Techno Electronic Music in the SUD group (*t = −2.961; p =.003*). Finally, no statistically significant differences were identified between the AUD and SUD groups concerning their preferences for other music genres (*p >.05*).

Among the variables that showed significant differences between the AUD and SUD groups, correlation analyses between age, educational status, and music preferences revealed a statistically significant weak positive correlation between the preference for Classical Turkish Music and age (*r =.263; p <.001*). Conversely, a statistically significant negative moderate correlation was identified between Hip-hop/Rap and age (*r = −.301; p <.001*). Additionally, a statistically significant positive correlation was found between Hip-hop/Rap music preference and educational status (Spearman’s ρ = 0.185, *p* <.001), indicating that individuals with higher educational levels tended to show greater preference for Hip-hop/Rap music. No correlations were found between age and preference for Techno Electronic Music, nor between educational status and preferences for Classical Turkish Music and Techno Electronic Music (*p* >.05).

The K-means clustering analysis conducted in this study to identify listener profiles based on music genre preferences resulted in three statistically significant clusters. Notably, the majority of individuals from the alcohol and substance group were predominantly classified within cluster 2. The values indicating the final cluster centres for each cluster concerning the music genre variables are summarized in Table 2.

**Table 2:** Final cluster centres by music types

Music Types	Cluster 1 (*n* = 53)	Cluster 2 (*n* = 246)	Cluster 3 (*n* = 52)
Turkish Folk Music	1	2	3
Turkish Pop Music	3	2	4
Classical Turkish Music	1	1	3
Arabesque/Fantasy Music	1	3	3
Foreign Pop Music	4	1	3
Anatolian Rock Music	2	0	3
Hip-hop/Rap Music	4	1	3
Classical Western Music	2	0	3
Rock/Metal Music	2	0	1
Techno Electronic Music	4	1	2
Jazz/Blues	1	0	1
Sufi Music	0	0	2
Latin Music	1	0	1
Flamenco Music	1	0	1
Tango Music	1	0	0
Fado	0	0	0
Greek Music	0	0	0
Indian Music	0	0	1
Far Eastern Music	1	0	1
African Music	1	0	0

Values represent mean scores (0–5) for each music genre within each cluster. Except for Fado (*p =.093*), differences in mean scores across clusters were statistically significant (ANOVA, *p* <.05). Interpretation of cluster profiles should focus on the pattern of means

Based on the findings in Table 2, the profile of each cluster is as follows.

### Cluster 1 (*n* = 53): High-Energy and Modern Preferences

This cluster is characterized by individuals who exhibit a pronounced affinity for contemporary and energetic music genres. The mean scores indicate a strong preference for Techno/Electronic (mean score = 4), Hip-hop/Rap (mean score = 4), and Foreign Pop music (mean score = 4). Moderate interest is observed in genres such as Anatolian Rock music (mean score = 2) and Classical Western Music (mean score = 2). Conversely, there exists a significantly low interest in traditional and local musical styles, including Turkish Folk Music (mean score = 1), Classical Turkish Music (mean score = 1), and Arabesque/Fantasy Music (mean score = 1). Additionally, interest in certain music styles, such as Fado, Greek, Indian, and African music, is minimal, reflected by a mean score of 0.

### Cluster 2 (*n* = 246): Preference for Arabesque/Fantasy Music and Limited Musical Taste

This cluster represents the largest segment of the sample. Members show moderate interest specifically in Arabesque/Fantasy music (mean score = 3/5), with some interest in Turkish Folk (mean = 2) and Turkish Pop (mean = 2). In contrast, interest in most other genres—including Western and ethnic music—is minimal (majority scoring 0–1).

### Cluster 3 (*n* = 52): Broad and Balanced Musical Tastes

Individuals within this cluster demonstrate a diverse and balanced appreciation for both traditional and modern musical genres. High levels of interest are indicated in Turkish Folk music (mean score = 3), Classical Turkish music (mean score = 3), Arabesque/Fantasy music (mean score = 3), Anatolian Rock music (mean score = 3), Foreign Pop music (mean score = 3), and Hip-hop/Rap (mean score = 3). Furthermore, there is notable inclination towards music encompassing different cultural backgrounds, such as Classical Western music (mean score = 3), Techno/Electronic (mean score = 2), and Sufi music (mean score = 2).

No statistically significant differences were observed among the three clusters regarding diagnosis (alcohol-substance), gender, and place of residence (*p* >.05). However, significant differences were identified concerning marital status and educational attainment (*p* <.05). Post hoc analyses revealed that Cluster 1 contained a significantly lower number of individuals with primary education compared to expected values, whereas the proportion of individuals with university education was significantly elevated (*p* <.0055). In contrast, Cluster 2 exhibited a significantly higher number of individuals with primary education than expected, while those with university education was underrepresented (*p* <.0055). Cluster 3 showed no differences in educational levels, with high school education levels remaining consistent across groups (*p* >.0055) **(**Table 3**)**.

**Table 3:** Comparison of patients’ sociodemographic characteristics according to clusters

		Cluster 1 (*n* = 53)	Cluster 2 (*n* = 246)	Cluster 3 (*n* = 52)	χ²	*p*
Diagnosis	Alcohol	13	72	12	*1.124*	*0.570*
	Substance	40	174	40		
Gender	Male	33	171	35	*1.072*	*0.585*
	Female	20	75	17		
Place of Residence	Rural	13	69	13	*0.406*	*0.816*
	Urban	40	177	39		
Marital Status	Married	21	123	31	*9.924*	0.042*
	Single	25	90	10		
	Divorced	7	33	11		
Educational Status	Primary	15	133	23	*18.867*	0.001**
	High school	26	96	21		
	University	12	17	8		
Economic Status	< 17k	24	109	20	*5.78*	*0.44*
	17k-34k	17	100	22		
	34k-51k	6	27	7		
	> 51k	6	10	3		

*Bonferroni correction was applied for the P value*,* and the significance level was accepted as 0.0055*

* *No significant results were found in the post hoc analysis (p >.0055)*

** In the post hoc analysis,* the expected values for clusters 1 and 2 were found to be elementary school (Z=−3.2*, *p =.0013; Z = 3.1,p =.0019) and university (Z = 3.1*,*p =.0019; Z=−3.4*,*p <.006)*,* respectively. No significant differences were found in the distribution of high school and cluster 3 compared to expected values (p >.0055)*

In a comparative analysis of the three clusters based on music preferences and personality traits, a statistically significant difference was observed in NS scores (*p* <.001). Specifically, Cluster 1 demonstrated considerably higher NS scores than both Cluster 2 and Cluster 3 (*p <.001*). With regard to C scores, a significant difference was also noted among the clusters (*p =.021*), with Cluster 3 exhibiting significantly higher C scores compared to Cluster 2. Although a statistically significant difference was identified in RD scores (*p =.046*), this finding did not remain significant following post hoc analyses. Additionally, no significant differences were found in the other temperament character scores (* >.05*). An examination of API scores and average ages across the clusters indicated no significant differences as well (*p >.05*) (Table 4).

**Table 4:** Comparison of clinical characteristics of patients according to clusters

	Cluster 1 Mean ± SD	Cluster 2 Mean ± SD	Cluster 3 Mean ± SD	F	*P*
NS	21.68 ± 4.61	19.03 ± 4.22	18.25 ± 4.98	*9.692*	0.000*
HA	17.09 ± 6.31	17.91 ± 4.99	16.13 ± 4.66	*2.763*	*0.064*
RD	12.81 ± 2.51	12.13 ± 2.61	12.96 ± 2.80	*3.110*	0.046**
P	4.32 ± 1.60	4.74 ± 1.77	4.85 ± 1.65	*1.518*	*0.221*
SD	22.15 ± 6.76	22.49 ± 6.06	22.71 ± 5.67	*0.114*	*0.892*
C	25.92 ± 6.10	24.31 ± 5.83	26.40 ± 4.95	*3.909*	0.021***
ST	20.36 ± 4.96	19.93 ± 5.39	21.06 ± 5.30	*1.015*	*0.364*
API Total	11.48 ± 3.63	10.78 ± 4.03	10.76 ± 3.72	*0.718*	*0.488*
Age	33.92 ± 7.84	35.41 ± 10.34	37.73 ± 9.16	*2.022*	*0.134*

Tukey HSD correction was applied to the p-value found in all post hoc tests

* In the post hoc analysis,* significant differences were found between Cluster 1 and Cluster 2 and between Cluster 1 and Cluster 3 (p <.001). No significant difference was found between Cluster 2 and Cluster 3 (p >.05)*

** No significant difference was found in the post hoc analysis (*p* >.05)

*** *Post hoc analysis revealed a significant difference between Cluster 2 and Cluster 3 (p =.046)*

## Discussion

The primary findings of the study indicate that individuals diagnosed with AUD and SUD exhibit limited musical preferences and show minimal interest in diverse musical genres. Furthermore, a correlation exists between certain personality traits and musical preferences, with individuals’ genre preferences varying according to their specific diagnosis (AUD versus SUD).

In the context of this study, participants were evaluated through a music preference survey and subsequently categorized into three distinct groups. A substantial proportion of AUD and SUD patients (*n* = 246) were identified as belonging to the Arabesque/Fantasy music-oriented and limited music taste category. This categorization suggests that these individuals possess a diminished interest in music overall, demonstrating a tendency to prefer only a narrow range of genres, such as Arabesque/Fantasy music. The Arabesque/Fantasy music genre, which emerged in Türkiye during the 1960 s, is characterized by thematic elements of pain, helplessness, separation, identity, and transformation. It is posited that this genre reflects the sociocultural identities of marginalized lower socioeconomic classes while simultaneously representing broader social realities. Given that Arabesque music frequently engages with themes of emotional suffering and social struggle, it has been interpreted as both a reflection and a reinforcement of patterns of alcohol and substance use within its cultural context [26, 27]. The Arabesque/Fantasy Music preferences of the patients participating in our study are unsurprising and consistent with the existing literature [28]. However, a notable finding is that these patients only listen to this type of music moderately frequently and generally show very little interest in music. This suggests that the decline in AUD and SUD patients’ interest in healthy lifestyle habits has expanded to include music listening [29, 30].

Our study reveals a notable relationship between individuals’ music preferences and specific temperament traits, namely NS, RD and C. In Cluster 1, individuals exhibited a higher NS temperament trait score compared to those in Clusters 2 and 3. This cluster includes individuals who favour high-energy and contemporary genres such as hip-hop/rap and techno/electronic music. These individuals demonstrate a pronounced tendency toward exploratory behaviour in response to new stimuli, characteristic of the Novelty Seeking trait. Supporting these findings, a study conducted in the USA indicated that individuals with a strong inclination toward innovation are drawn to genres like rap, punk, heavy metal, and reggae [17]. A study conducted in China in 2020 revealed that individuals seeking greater innovation tend to prefer genres like electronic dance music, which provides constant stimulation and appeals to those desiring new social experiences during nightlife [31]. Additionally, a study from 2017 identified openness to experience as the personality dimension most closely linked to music preference [16]. When these findings are evaluated alongside our own research, it becomes evident that individuals with a high need for sensation (NS) are more inclined to favour high-energy music genres such as hip-hop/rap and techno electronic music.

In comparing personality traits, Cluster 3 exhibited a higher Cooperativeness (C) score than Cluster 2. This cluster comprises individuals with diverse and balanced musical tastes who demonstrate greater attributes of empathy, kindness, social acceptance, and helpfulness. Patients within Cluster 3 expressed a keen interest in various music styles, including Turkish folk music, Classical Turkish Music, Arabesque/Fantasy music, Anatolian rock/original, and Foreign Pop Music. A study analysing the correlation between music preferences and personality traits among university students in Pakistan found that characteristics such as extroversion, agreeableness, and cooperativeness were positively associated with a wide array of music types. This includes intense and rebellious music, optimistic and traditional music, as well as reflective and complex music, aligning with our findings [32]. Notably, the significant differences in RD temperament scores across groups disappeared following post hoc analysis.

No significant differences were observed among the three clusters concerning sociodemographic characteristics, including age, gender, marital status, place of residence, and income level. This lack of variation precluded these variables from being identified as confounding factors. Notably, in terms of educational status, the proportion of individuals with only primary school education was higher in Cluster 2. This finding suggests that a lower level of education may correlate with reduced engagement in music consumption and a narrower range of musical interests. Supporting this interpretation, research conducted by Favaro and Frateschi indicates that individuals with higher levels of educational attainment are likely to appreciate a broader spectrum of music genres, contrasting with those from lower educational backgrounds who typically exhibit more limited preferences. This observation aligns with the findings of the present study [33].

When analysing the data by grouping patients into alcohol users and substance users, comparable trends were identified regarding gender, marital status, and economic status, which similarly prevented these characteristics from being classified as confounding factors. However, an age comparison revealed that the average age of patients in the Alcohol group was significantly higher than that of the Substance group. This finding is consistent with previous research examining age-related patterns in substance use, which indicates that younger individuals are more inclined to experiment with various substances, while older individuals display a preference for alcohol over other substances [34]. Furthermore, another study has identified younger age as a risk factor for SUD [8].

In assessing educational status among the two groups, there was a higher percentage of university graduates within the Alcohol group. This disparity in educational attainment may be attributed to the relatively lower average age of patients within the Substance group, resulting in fewer individuals who have progressed to or completed university education. Additionally, correlation analyses demonstrated a preference for Classical Turkish music among older patients, while younger individuals exhibited a greater affinity for hip hop and rap genres. A study investigating age-related trends in music preferences among adults has indicated that variations in characteristics such as timbre, dynamics, and tone clarity are correlated with age [35]. Taken together, these findings suggest that music preferences may undergo significant changes as individuals age.

In our study, we found that patients in the Alcohol group exhibited a stronger preference for Classical Turkish music compared to those in the Substance group, who favoured Hip-hop/Rap and Techno Electronic music genres. This aligns with previous research examining the connection between alcohol and cannabis use and music preferences, which indicated that cannabis users tend to prefer hip-hop/rap, while alcohol users lean towards local and traditional music [36]. Various studies have highlighted that the type of substance use is influenced by musical genres, noting that listeners of rap and hip-hop, rock, and dance/electronic music are particularly prone to substance use [36–38]. It has been specifically noted that rap and hip-hop music frequently contain extensive references to substance use, and increased exposure to this genre correlates with higher rates of substance use among listeners [19, 39, 40]. Furthermore, research conducted on Dutch adolescents revealed that classical and traditional music is associated with lower substance use, whereas techno music correlates with increased usage [41]. Additionally, it has been pointed out that most references to alcohol and drug use in music are presented positively, rarely addressing the negative consequences of consumption, and that many songs glorify drugs, tobacco, and alcohol, linking their use to favourable images of success, wealth, charm, and socialization [42, 43]. 

Finally, from a clinical perspective, these findings may hold significance, as music preferences reflecting personality traits could inform more individualized approaches during both the early motivational and later rehabilitative phases of addiction treatment. Incorporating preferred music genres into therapeutic interventions may enhance engagement, emotional regulation, and therapeutic alliance. This interpretation aligns with previous evidence suggesting that music-based therapies can reduce craving, improve emotional stability, and strengthen treatment adherence in individuals with substance use disorders [44–46].

This investigation, characterized by a substantial sample size, makes significant contributions to the existing body of literature by exploring the interplay between personality traits, musical preferences, and disorders profiles among individuals diagnosed with AUD and SUD. However, the cross-sectional design employed herein imposes limitations on causal inferences. Moreover, the reliance on self-report measures may introduce the potential for social desirability bias, thereby affecting the authenticity of the data collected. Additionally, due to the study’s localization within a single centre, the extrapolation of findings to diverse sociocultural contexts may be restricted. Although the MPQ scale used in our study was formulated with an awareness of the Turkish cultural landscape, the lack of psychometric standardization, such as validity-reliability studies, is a significant limitation. Notwithstanding these limitations, the study is bolstered by its large sample size, comprehensive data set, and the application of various statistical analyses, which collectively enhance the reliability and validity of the findings. In this regard, the research serves as a distinctive contribution to the field of addiction by concurrently addressing the domains of musical preferences and personality structures.

## Conclusion

In conclusion, individuals diagnosed with AUD or SUD demonstrate a generally constrained interest in a diverse array of music genres, with a notable predominance for styles such as Arabesque/Fantasy music. This study’s findings reveal a significant correlation between music preferences and personality characteristics, specifically novelty seeking and cooperativeness. Individuals exhibiting a high novelty-seeking temperament tend to favour high-energy music genres, including hip-hop/rap and techno/electronic music. Conversely, those who display a higher degree of cooperativeness are more likely to prefer traditional, broad, and balanced musical styles. These results suggest that personality traits may play role in shaping musical tastes. Additionally, factors such as age and diagnostic classification (AUD versus SUD) have emerged as pertinent influences on musical taste. This suggests a complex interplay among psychological traits, clinical profiles, and the expression of culture through music.

## Data Availability

The data sets used and/or analysed during the current study are available from the corresponding author on reasonable request.

## Abbreviations

SUD: Substance use disorders
AUD: Alcohol use disorders
WHO: World Health Organization’s
UNODC: United Nations Office on Drugs and Crime
IDC: Inpatient detoxification centre
MPQ: Music preferences questionnaire
TCI: Temperament character inventory
API: Addiction profile index
NS: Novelty seeking
HA: Harm avoidance
RD: Reward dependence
P: Persistence
SD: Self-directedness
C: Cooperativeness
ST: Self-transcendence
